# Longitudinal alterations in the urinary virome of kidney transplant recipients are influenced by BK viremia and patient sex

**DOI:** 10.1128/spectrum.04055-23

**Published:** 2024-06-25

**Authors:** Rabia Maqsood, Lily I. Wu, Daniel C. Brennan, Efrem S. Lim

**Affiliations:** 1 Center for Fundamental and Applied Microbiomics, The Biodesign Institute, Arizona State University, Tempe, Arizona, USA; 2 Department of Medicine, Division of Nephrology, Johns Hopkins University School of Medicine, Baltimore, Maryland, USA; 3 School of Life Sciences, Arizona State University, Tempe, Arizona, USA; University of Texas Southwestern Medical Center, Dallas, Texas, USA

**Keywords:** urinary virome, longitudinal, kidney transplantation, BK, patient sex difference, urotype (urine community states)

## Abstract

Little is known about the urinary virome and how it interacts with the host, particularly in renal transplant diseases. Using metagenomic sequencing, we characterized the urinary virome of 23 kidney transplant recipients longitudinally (11 BKV+ patients and 12 BKV− patients). We applied linear mixed effects models, PERMANOVA, k-means clustering, and MaAsLin2 algorithms to determine virome signatures associated with post-transplant time, BK viremia status, and patient sex. We found that the richness and alpha diversity of urinary virome were significantly different in renal transplant recipients with BKV+ over time in comparison to BKV− (richness *P* = 0.012, alpha *P* < 0.0001). Female BKV− patients had significantly higher virome richness than males (*P* = 0.0063). Virome beta diversity was significantly different between patients by BKV status (*P* < 0.001). Additionally, we identified underlying interactions between patient sex and BKV status, in terms of virome beta diversity (*P* = 0.008). BK polyomavirus infections were primarily of subtypes IA, IB1, and IB2. The non-BK dominant samples clustered into six urinary virome community states. BKV− samples had more anelloviruses than BKV+ samples though this difference was not statistically significant. Lastly, we identified specific viruses, associated with BKV+ and time in our samples. Our results indicate that dynamic alterations in the urinary virome over the post-transplant period in kidney transplant recipients can be shaped by BK viremia and patient sex. These findings advance our fundamental understanding of the urinary virome and support a new line of investigation in renal disease and transplantation.

**IMPORTANCE:**

The urinary microbiome is increasingly implicated in renal health and disease. While most research focuses on bacteria communities of the microbiome, factors that influence the urinary virome are not understood. Here, we investigated the urinary virome of 23 adult kidney transplant recipients longitudinally over 14 weeks post-transplant. We show that alterations in the urinary virome are associated with kidney transplant recipients with BK polyomavirus viremia that can lead to BK nephropathy and allograft rejection. By modeling the temporal dynamics post-transplant, we delineated specific profiles of the urinary virome associated with patient sex and urinary community states. These findings reveal fundamental aspects of the urinary virome that can be leveraged to better manage kidney diseases.

## INTRODUCTION

The microbiome includes a diverse community of viruses (virome), bacteria (bacterial microbiome), and other microorganisms that interact with the immune system and contribute to an individual’s health. Growing evidence indicates that the microbiome is implicated in transplantation and immunosuppressive therapy as alterations in the microbiome have been associated with acute and chronic rejection in solid organ transplant recipients (1, 2). However, such detailed studies have not been conducted for the urinary virome. Historically, urine from healthy individuals has been considered sterile. However, a few studies in recent years have identified unique virome found in urine (3, 4). Viruses commonly detected in urine include BK polyomavirus, human papillomaviruses, anelloviruses, mastadenoviruses, and herpesviruses (3–7). One study found that urinary viruses differ by patient sex in children with males having more abundance of *Mastadenovirus and human Mastadenovirus* present in their urine in comparison to females, but other studies have not yet found an association by patient sex in adults (3–5).

Studies of the urinary virome are limited; however, there is growing evidence of bacteriophages and eukaryotic viruses found to be present in urine (3, 6–8). BK polyomavirus (BKPyV) and JC polyomavirus, anelloviruses, human papillomaviruses, herpesviruses, and adenovirus have been identified in urine by metagenomic sequencing and mass spectrometry studies (3, 7, 9). Studies monitoring plasma levels observed an inverse association between TTV (anellovirus) and allograft rejection—whereby decreased TTV levels were associated with an increased risk of allograft rejection in kidney transplant recipients (10). However, other studies did not find this association when analyzing the urine of transplant recipients (11, 12), suggesting a more complex relationship in transplantation. Most urinary virome studies to date have been cross-sectional. Studies of other body sites, such as the gut, indicate that intraindividual variation in the virome over time within a person can change with disease (13). Thus, longitudinal studies of the urinary virome are needed to better understand temporal dynamics within an individual and how it changes in health and disease.

BKPyV infection and nephropathy are significant causes of renal dysfunction and allograft loss in renal transplant recipients (14, 15). In this study, we investigated the longitudinal urinary DNA virome of 23 kidney transplant recipients’ post-transplantation using viral metagenomic sequencing. Since BK viremia is implicated with BK polyomavirus-associated nephropathy and renal allograft failure, we compared the urinary virome of transplant recipients with BK viremia or without BK viremia. We found significant intrapersonal variation in the urinary virome over time that differed by BK viremia status. This study fills the knowledge gap by elucidating the temporal dynamics of the urinary virome in kidney transplant patients.

## RESULTS

**Fig 1:**
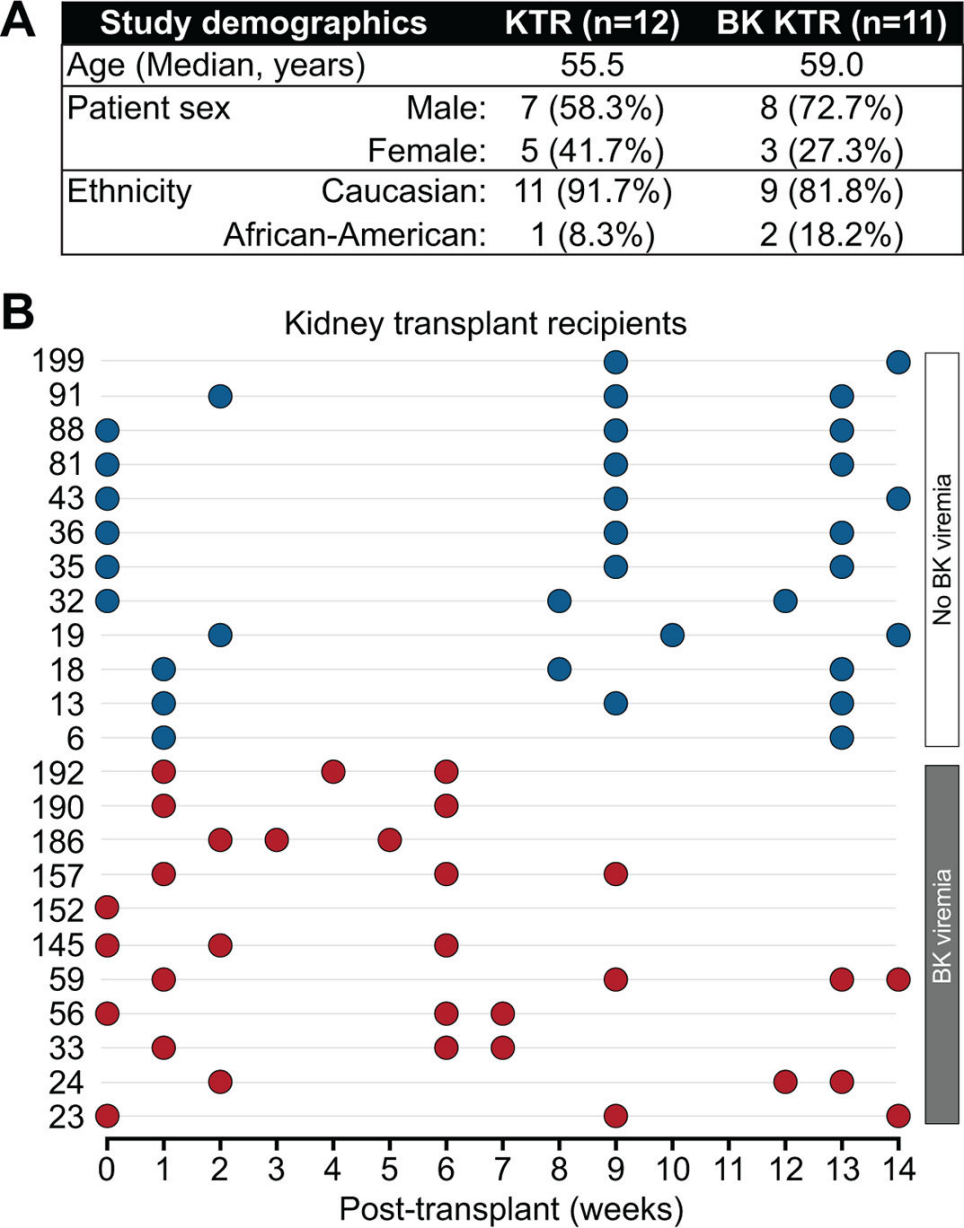
Cohort study design. (**A**) Cohort of 23 kidney transplant recipients in this study is shown of corresponding urine specimen timepoints and BK viremia status. (**B**) Urine samples at week of sample collection are shown per kidney transplant patient.

To understand the role of the urinary virome in renal transplant and BK disease, we performed metagenomic sequencing to profile DNA viral communities of 65 urine specimens from 23 adult kidney transplant recipients (Missouri, USA). This study is a retrospective analysis of urine sample collected between 2000 and 2002 from kidney transplant recipient patients enrolled in a prospective study to prevent BK nephropathy (16). Eleven individuals were clinically diagnosed with BK viremia (BKV+), the hallmark of progression towards BK virus-associated nephropathy (BKVAN), and 12 recipients without BK viremia (BKV−). Fifteen transplant recipients were male (72.7% of BK viremia recipients) and eight were female (27.3% of BK viremia recipients). The median age in this study was 57 years, 3 African American patients (13%) and 20 White patients (87.0%) (Fig. 1A). Repeated urine specimens were collected from transplant recipients between 0 and 14 weeks post-transplantation to assess changes over time (Fig. 1B). The most abundant viral contigs were from viral families *Polyomoviridae* (31%), *Anelloviridae* (10%), *Circoviridae* (9%), and *Peduoviridae* (4). There were also a large proportion of the contigs that were unclassified bacteriophages from class *Caudoviricetes* (28%) or were unclassified viruses (9%).

### Urinary virome signatures associated with BK viremia

**Fig 2:**
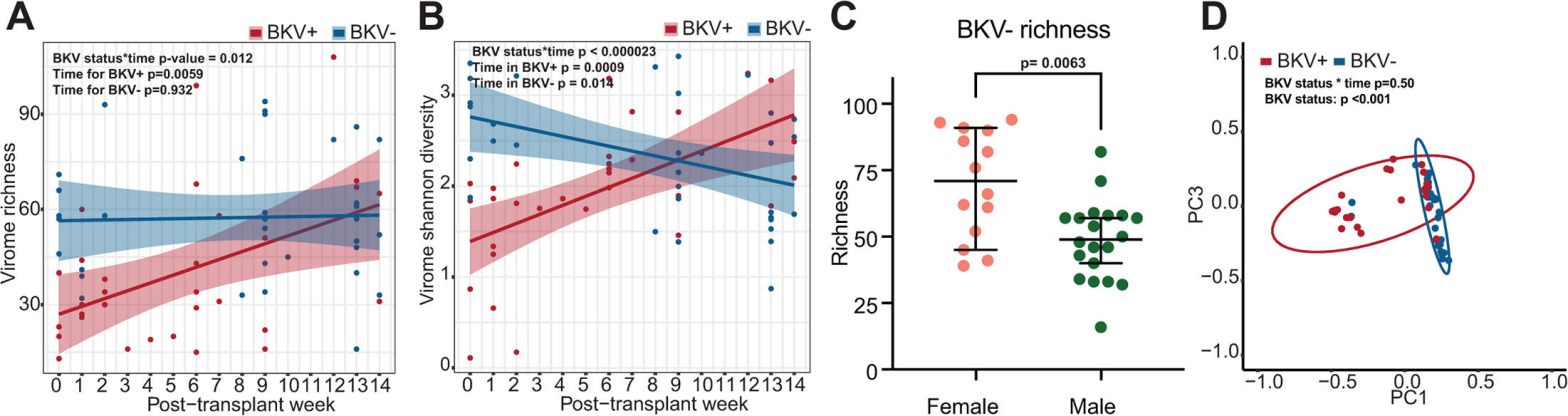
Urinary virome analysis for all samples. (**A**) Linear plot of virome species richness over post-transplant week for BKV− and BKV+ samples. Statistical significance was assessed by linear mixed effect model. (**B**) Linear plot of virome alpha diversity (Shannon) over post-transplant week for BKV− and BKV+ samples. Statistical significance was assessed by linear mixed effect model. (**C**) Viral richness of females and males in BKV− group. Statistical significance was assessed by linear mixed effect model. (**D**) Beta diversity PCoA analysis of weighted Bray-Curtis dissimilarity distance is shown. BKV status is indicated by colors. Statistical significance was assessed by PERMANOVA.

We first wanted to assess if there were interactions between the metadata of patient sex with BKV status, patient sex with post-transplant time, and BKV status with time for virome richness, alpha and beta diversity. We applied LME (richness and alpha diversity) and PERMANOVA (beta diversity) models but did not find statistically significant interactions between patient sex and BKV status or patient sex and time for virome richness, alpha or beta diversity (*P*-values of patient sex*BKV/patient sex*time; richness *P* = 0.24/0.57, alpha diversity *P* = 0.96/0.065, beta diversity *P* = 0.096/0.60). We found significant interactions between BKV status and time for richness and alpha diversity, but not beta diversity (richness *P* = 0.012, alpha diversity *P* < 2.28E-05, beta diversity *P* = 0.50; Fig. 2A and B). Since the models indicate that there is an interaction between BKV status and post-transplant time, we stratified data by BKV status (BKV+ and BKV−) for richness and alpha diversity. When stratified, we found the richness in the BKV+ to be significantly increasing over time; however, the BKV− richness did not change throughout the 14 weeks (BKV+ *P* = 0.0059, BKV− *P* = 0.932; Fig. 2A). Instead, the richness in the BKV- samples was significantly different by patient sex, with the females having more richness in urine than males (*P* = 0.0063; Fig. 2C). Interestingly, virome alpha diversity increased significantly over time in BKV+ patients, but this was reversed in BKV− patients (BKV+ *P* = 0.0009, BKV− *P* = 0.014; Fig. 2B). Virome beta diversity clustered distinctly by BKV status (*P* = 0.001; Fig. 2D). Taken together, this suggests that the substantial changes in the urinary virome are associated with BKV in renal transplant recipients.

**Fig 3:**
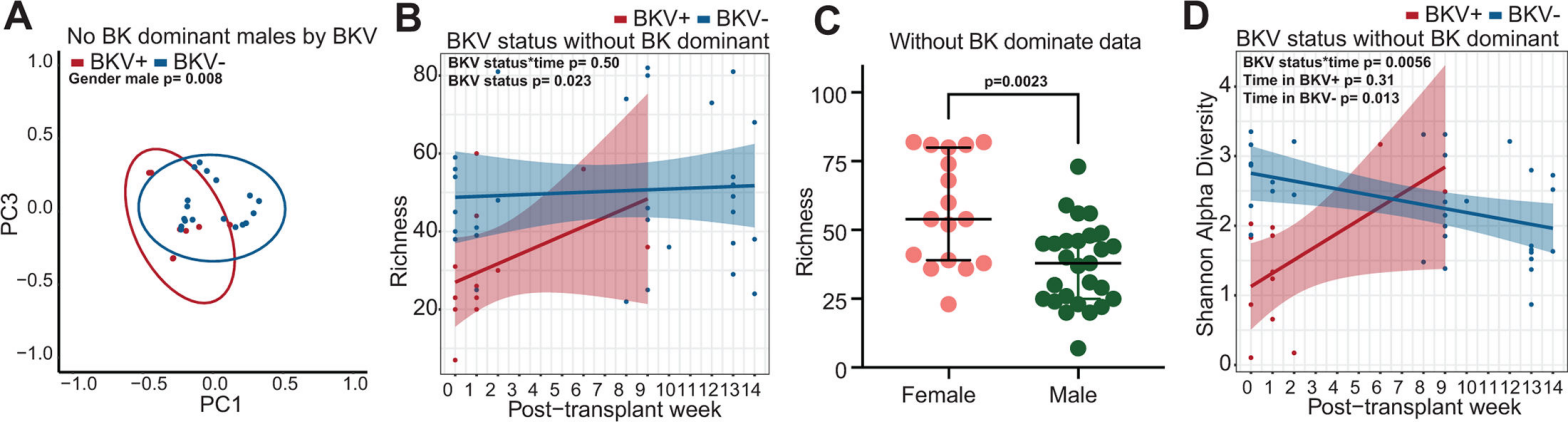
Urinary virome analysis for samples stratified by BK polyomavirus dominance. (**A**) Beta diversity PCoA analysis of weighted Bray-Curtis dissimilarity distance is shown for males. BKV status is indicated by colors. Statistical significance was assessed by PERMANOVA. (**B**) Linear plot of virome species richness over post-transplant week for BKV− and BKV+ samples without BK polyomavirus dominance. Statistical significance was assessed by linear mixed effect model. (**C**) Viral richness of females and males in samples without BK Polyomavirus dominance. Statistical significance was assessed by linear mixed effect model. (**D**) Linear plot of virome alpha diversity (Shannon) over post-transplant week for BKV− and BKV+ samples without BK polyomavirus dominance. Statistical significance was assessed by linear mixed effect model.

Since BK polyomavirus infection itself is linked to the disease, we sought to control for this by removing BK Polyomavirus counts (i.e., BKV dominated signals) and rerun the LME and PERMANOVA models above. By excluding BKV, this would ascertain whether there were other *bona fide* urinary virome signatures. Beta diversity models showed that there was an interaction between patient sex and BKV status (*P* = 0.033). When stratified by patient sex, we found that the virome beta diversity differed significantly by BKV status in males but not females (female BKV status *P* = 0.834, male BKV status *P* = 0.008; Fig. 3A). Similarly, virome richness differed by BKV status as well as by patient sex. The urinary virome richness in females was higher than males and was dependent on BKV status (patient sex *P* = 0.0023, BKV status *P* = 0.023; Fig. 3B and C). Virome alpha diversity was still significant for the interaction between BKV status and time. The opposing trajectories in virome alpha diversity were still consistent, showing that BKV− significantly decreasing over time (*P* = 0.013) and BKV+ increasing over time though this was no longer statistically significant (*P* = 0.31) (Fig. 3D). Overall, we found significant differences in the virome by BKV status of the kidney transplant patients.

### Urinary virome community status

**Fig 4:**
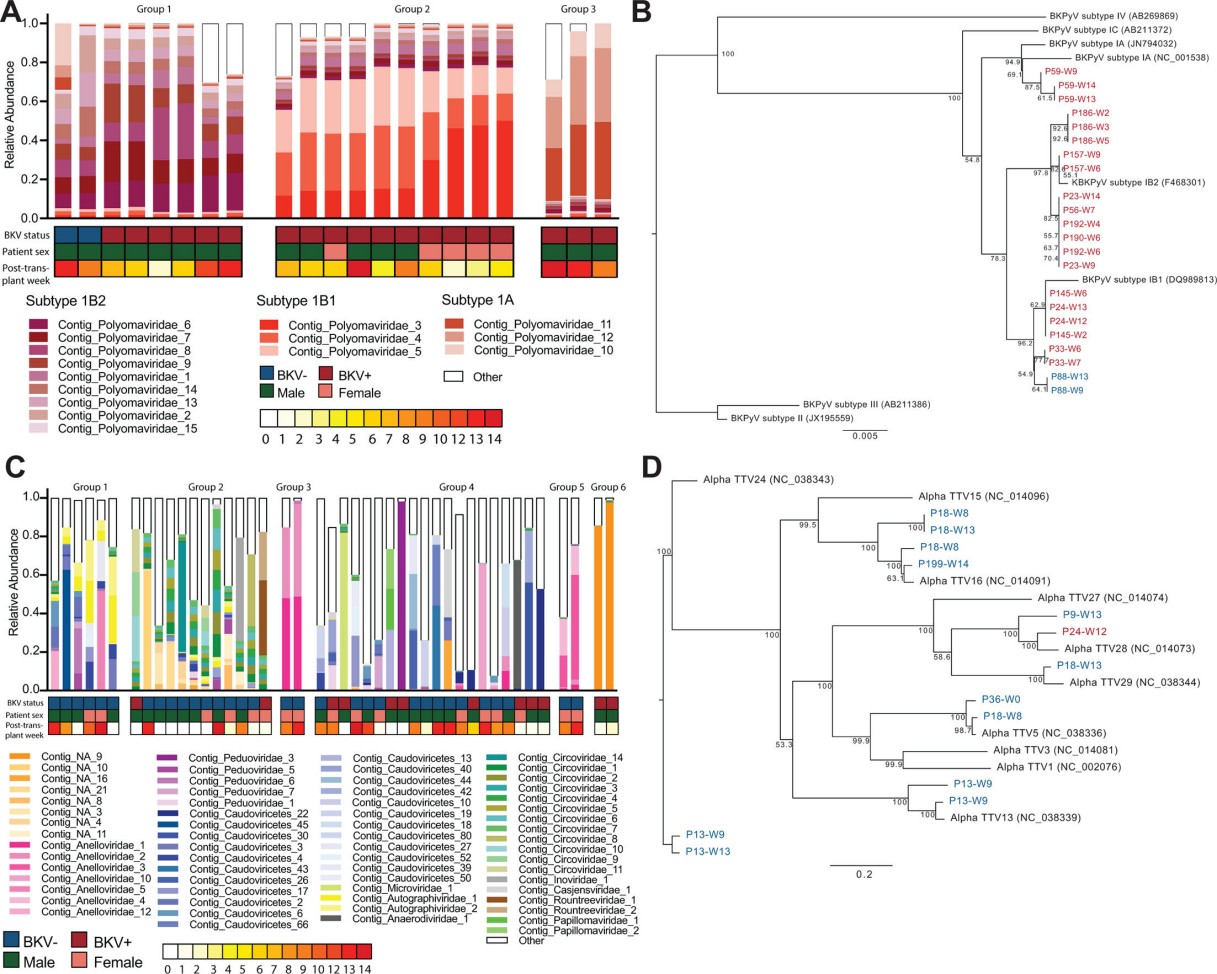
K-means clustering of samples with or without BK polyomavirus dominance. (**A**) K-means groups of samples with BKV dominance using weighted bray-curtis distances. Relative abundance of top contigs shown for each group. (**B**) Phylogenetic tree of BK Polyomavirus VP1 gene. BKV+ patients are shown in red, BKV− patients are shown in blue. (**C**) K-means groups of samples without BKV dominance using weighted bray-curtis distances. Relative abundance of top contigs shown for each group. (**D**) Phylogenetic tree of alpha torque teno virus (anellovirus) ORF1 gene.

We next characterized the urinary virome by community state profiles. Given the BKV dominance in some samples, we clustered them separately from the rest. We found that BK dominant viromes clustered into three distinct clusters that were made up of BK polyomavirus contigs specific to their cluster (Fig. 4A). Since there are four main subtypes of BK polyomavirus (subtypes I, II, III, and IV), we hypothesized that the clusters were driven by BKV subtypes. To test this, we performed a phylogenetic analysis on the VP1 region which can be used to classify subtypes from our samples and reference sequences. We found that community state group 1 samples were comprised of BK subtype IB2, group 2 contigs were from subtype IB1 and group three contigs were from subtype IA. Furthermore, the BKV sequences from the same individual at multiple different times were phylogenetically more closely related to one another consistent with a chronic BK infection (Fig. 4B).

In the samples without BK dominant samples, we found six distinct urinary community states (Fig. 4C; Fig. S1A). Group 1 was most abundant in five *Caudoviricetes* contigs (between 13% and 3%, sum 29%), two *Anelloviridae* contigs (10% and 4%, sum 14%) two *Autographiviridae* contigs (both 8%, sum 16%), and one *Peduoviridae* contig (5%). Group 2 was highly abundant and prevalent in 10 *Circoviridae* contigs (5%–3%, sum 33%), one unclassified contig (5%), one *Inoviridae* contig (4%), and one *Rountreeviridae* contig (3%). Group 3 was dominated by two *Anelloviridae* contigs (48% and 42%, sum 91%). Group 4 was abundant in one *Peduoviridae* (5%), one *Microviridae* contigs (4%), two *Caudoviricetes* contigs (both 3%, sum 6%), three *Anelloviridae* contigs (all 3%, sum 9%), and one *Anaerodiviridae* contig (3%). Group 5 was abundant in two *Anelloviridae* contigs (35% and 17%, sum 53%), different from group 3 *Anelloviridae* contigs. Group 6 was dominate one unclassified contig (92%). We found no significant association between groups and BKV status, patient sex, or time by multinomial logit models.

Plasma anellovirus loads have been implicated in immunosuppression and allograft rejection in solid organ transplant recipients (17–19). We found that the counts of anelloviridae reads in urine was significantly higher in BKV− specimens compared to BKV+ in the sequencing data set (*P* = 0.0017) (Fig. S1B). We also found the females to have significantly more anelloviridae in their urine in comparison to males (*P* = 0.0038, Fig. S1C). Using qPCR assays to quantify anellovirus load in the urine specimens, we detected urine anellovirus load at higher median levels in BKV− specimens (median 15,786 copies/mL) than compared to BKV+ specimens (median 981.9 copies/mL). However, the difference was not statistically significant (*P* = 0.149) (Fig. S1D). When comparing the anellovirus qPCR load by patient sex, we found the females (median 28,523 copies/mL) to have significantly higher anellovirus than males (117.8 copies/mL, *P* = 0.0002, Fig. S1E).

Because anellovirus contigs were found in many samples with no individual cluster associated with all contigs, we decided to create a phylogenetic tree of the anelloviruses from our samples. We found that anellovirus species TTV5, TTV13, TTV16, TTV28, and TTV29 were most closely related to the anellovirus contigs from our samples. We also found that two patients (13, 18), both females from control group, had multiple analogisms species throughout their time points in this study (Fig. 4D).

### Differential signatures of the urinary virome

**Fig 5:**
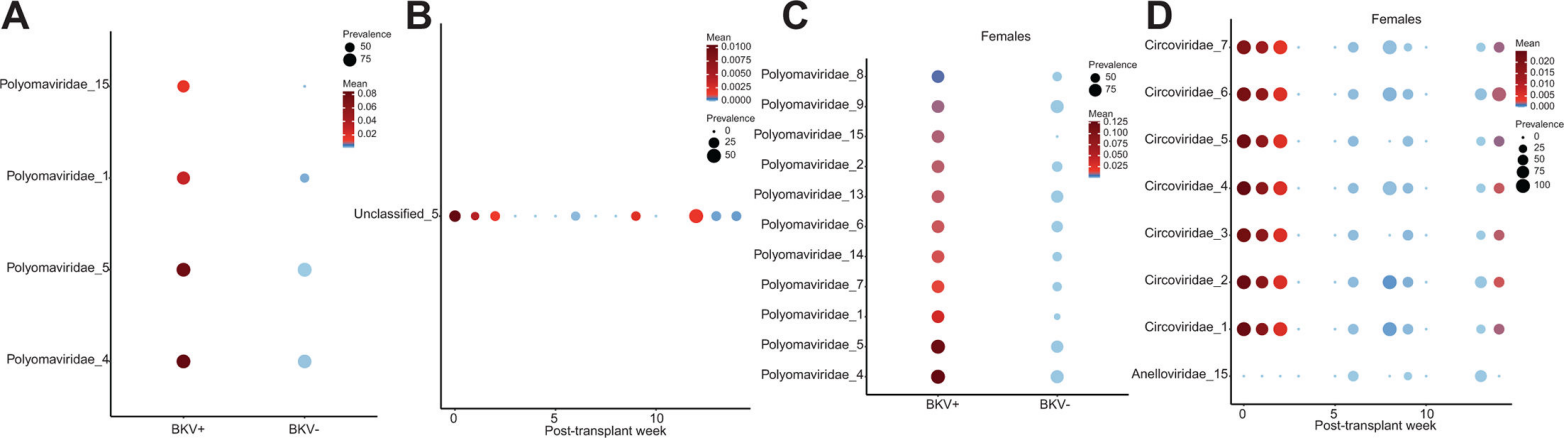
Differential analysis of viruses by BKV status, patient sex, and post-transplant week. (**A**) Differential polyomoviridae contigs by BKV status in all samples. (**B**) Differential unclassified contig by post-transplant week. (**C**) Differential polyomoviridae contigs by BKV status in females. (**D**) Differential Anelloviridae and Ciroviridae contigs by post-transplant week in females.

We next wanted to determine if there were specific viruses associated with BKV status, patient sex, or time. When looking all the data together, we found four *Polyomavirus* contigs to be the most important factor differentiating by BKV status (*P*-values between 0.0002 and 0.001, Fig. 5A). We also found one unclassified contig to be important by time, with it being high in abundance and prevalence in samples before transplant and then decreasing over the next few weeks following transplant and then seeming to recover at week 12 onward (*P* = 0.003, Fig. 5B). We also tried to run masslin2 on data stratified by BKV dominant and non-dominant, but we did not find anything significant in either group by BKV status, patient sex, or time. We then stratified the data by patient sex and found no differential contigs by BKV status, patient sex, or time in males. However, 11 *Polyomaviridae* contigs were found to be differentiating the females by BKV status (*P*-values between 0.0003 and 0.009, Fig. 5C). We also found seven *Circoviridae* contigs (*P*-values between 0.0031 and 0.004) and one *Anelloviridae* contig (*P* = 0.02) differentiating the females by time (Fig. 5D). *Circoviridae* contigs were high in abundance and prevalence pre-transplant and then started to decrease over the weeks following transplant and then finally seem to be recovering toward week 14. Taken together, *Polyomaviridae* contigs differentiated samples by BKV stats as expected, and we also found contigs, mainly *Circoviridae*, which seem to be decrease following transplant and recover towards week 14.

## DISCUSSION

Here, we conducted an in-depth metagenomic sequencing investigation of the urinary virome of kidney transplant patients and identified viral changes associated with BK polyomavirus viremia, patient sex, and time.

Factors that influence the urinary virome are not well understood (3, 7). Our results indicate that alterations in the urinary virome occur during the 14 weeks post-transplant period of renal transplant recipients. While the precise mechanisms responsible for these changes are not known, studies observed gut bacterial microbiome and virome dysbiosis during the post-transplant period in kidney transplant recipients (7, 9, 20, 21). Plasma virome of transplant patients has also been shown to be influenced by pharmacologic perturbation and immune suppression in individuals (17, 22). Disease-associated states (e.g., BK viremia) can impact trajectory of the urinary virome in transplant recipients even when controlling for the longitudinal changes associated with post-transplant. Non-invasive biomarkers of renal disease such as chemokines (e.g., CXCL9, CXCL10) and donor-derived cell-free DNA are increasingly being considered to improve diagnosis and predict allograft rejection (23, 24). Similarly, the urinary microbiota could also be used to monitor health and disease. We also identified patient sex-specific changes of the urinary virome. The preponderance of evidence for urinary microbiome patient sex differences stem from studies of bacteria in the urinary tract (3, 25, 26). Unlike another previous studying urinary virome in UTI patients, this study demonstrates that specific differences in the urinary virome are associated with patient sex (3). In our study, the females had a higher richness than males when not including BKV status or data. Thus, this highlights that patient sex differences contribute to the urinary microbiome community structure as well; however, future studies with healthy and diseased individuals are needed to understand the differences by patient sex.

A previous study found bacteriophages dominated the urinary virome, whereas we found eukaryotic viruses such as anellovirus and circoviruses along with bacteriophages (3). This could be due to differences in sample processing methodologies (e.g., cesium chloride density gradient ultracentrifugation). Kidney transplant recipients with BK viremia had lower levels of anellovirus (torque teno virus) in urine compared to kidney transplant recipients without BK viremia. Growing evidence implicates anelloviruses in host functional immunity. Decreased plasma anellovirus load is associated with graft rejection, while increased plasma anellovirus load is associated with over-immunosuppression (17–19). Previous studies that analyzed the urine of liver transplant or kidney transplant patients found anellovirus load was higher in recipients BKV- patients, though they too did not find significant associations, consistent with our findings (7, 11). Although it is not known whether anelloviruses are compartmentalized within plasma and urine, our findings indicate that the host immune responses implicated in BK nephropathy is reflected in the urinary virome alterations. Furthermore, this opens new opportunities to investigate the role of the urinary virome in other solid organ transplantations.

Our results underscore the complex nature of the urinary microbiota. A limitation of this study was that healthy, non-transplant individuals were not assessed to characterize baseline healthy states of the urinary virome. Future studies will need to determine how immunosuppression levels impact the urinary virome. Our findings advance the understanding of the temporal dynamics of the urinary virome in kidney transplant recipients and the interactions with the bacterial microbiome.

## MATERIALS AND METHODS

### Study cohort

This study was approved by the Washington University School of Medicine in St. Louis Institutional Review Board and by the Arizona State University Institutional Review Board. Adult kidney transplant recipients at Washington University Medical Center in St. Louis were enrolled and consented to the study. Recipients were previously clinically screened for BK viremia (16). Sustained BK viremia was defined as two or more consecutive plasma samples spanning three or more weeks. Reporting race and ethnicity in this study was mandated by the US National Institutes of Health (NIH), obtained from the study database.

### Virome sequencing and analysis

Midstream clean catch urine specimens were collected between 2000 and 2002 from kidney transplant recipient patients enrolled in prospective study to receive FK506 (16) and stored at −80°C. One milliliter aliquots of urine specimens was processed in 2016 byfiltering through a 0.45-µm membrane, and total nucleic acid was extracted from the filtrate on the COBAS Ampliprep instrument (Roche) as previously described (27). Multiple displacement amplification of total nucleic acid was performed with GenomiPhi v2 polymerase. Pooled libraries were sequenced on the Illumina MiSeq platform (2 × 250 v2 paired-end reads). Specimens were randomized by a random number generator for nucleic acid extraction, amplification, and next-generation sequencing.

Illumina MiSeq sequencing paired end reads (2 × 250), an average of 444,592 ± 238,190 DNA viral metagenomic reads, were quality filtered using BBtools [bbduk.sh for quality and length filter, bbmap.sh to remove human (GRCh38) and phix174 reads, dedupe.sh for removing duplicate reads, and bbmerge.sh to merge overlapping reads]. Quality filtered paired reads for each sample were used to build contigs with metaSPAdes for each sample (DNA urine sample *n* = 72, control *n* = 3) (28). We then removed human contigs using bowtie2 (101,487 DNA contigs), clustered the remaining contigs via cd-hit (69,815 DNA contigs), and then finally filtered by minimum contig length of 1,000 for DNA bp (17,883 DNA contigs) (29, 30). Theses contigs were then input into Cenote-Taker 2, VirSorter 2, and tblastx/blastn (NCBI NT database) to identify viral contig candidates (31–34). After the contigs initially passed each tool, we then used checkV for a final contig quality filtering and provirus screening (35). We used contigs with at least medium quality and those which had more viral contigs then host contigs as well as all proviruses identified, as per checkV. This gave us a total of (752 viral DNA contigs).

The viral contigs were then used to run blastx against viral RefSeq+ Neighboring sequences database (downloaded 2023). Using blastx output, we then used taxonomizr to assign taxonomy to each contig (family level) (34, 36). The final DNA contigs were then used as databases for which all urine sample QC reads were mapped against to get DNA virome matrix for all subsequent analysis using bowtie2. We used R package decontam and removed the called contaminants at threshold 0.25 for DNA viruses and used RPKM (reads per kilobase million) to normalize by contig length.

### LME and PERMANOVA models

To account for repeated measurements per person, we computed linear mixed-effects (LME) models, using R package nlme (version 3.1–148), to compare changes in richness and alpha diversity across longitudinal urine samples. Unweighted and weighted bray-curtis distances were compared using vegan, permute, and Adonis in R to compare changes in beta diversity across metadata using PERMANOVA. BKV status, patient sex, and time were included in the LME and Adonis models to account for possible confounding. The models first looked for interactions between the metadata (patient sex*BKV status, patient sex*time, BVK status*time), after which, if any interactions were significant, we would stratify by metadata and ran models for analysis. We first used all our data and samples together and ran the models, however, to take into consideration the differences being due to BKV dominating the urine of BKV+ patients, we also stratified the data into BKV+ dominate samples, and BKV+ dominate removed samples and re-ran the models to see differences across richness, alpha diversity, and beta diversity.

### Urinary community states analysis

To obtain the community states present in the urine samples of this study, we clustered our weighted beta diversity distance matrix with k-means method for the BKV dominate stratified data. We first used the R package factoextra (version 1.0.7) to determine the optimal number of clusters and then used stats function k-means to cluster the relative abundance at bacterial species level into three grounds for BKV dominate samples and six groups for the samples without BKV dominance. To determine associations between community states and time, BKV staus, and patient sex, we used R package mclogit (version 0.8.7.2) to perform multinomial logit models with random effects for patient ids; Benjamini-Hochberg method was used to correct for multiple comparisons for the mclogit results.

### Phylogenetic analysis

We build *de novo* BK polyomavirus genomes from our samples using QC reads mapped to BK polyomavirus in Geneious Prime (2023.0.4). BK polyomavirus has four many subtypes (I, II, III, and IV), with subtype 1 being further classified into four types (IA, IB1, IB2, and IC). We aligned our urine sample VP1 nucleotide sequences and references sequences from each subtype (AB269869, AB211372, JN94032, NC_001538, KF468301, DQ989813) using Geneious. After which, we built a neighbor-joining consensus tree, with a 1,000 bootstrap for confidence.

We built *de novo* anelloviruses from our samples with QC reads that mapped to TTV viruses. Our anellovirus genomes from urine all had close hits to alpha teno torque viruses, and therefore, we only used alpha anellovirus reference sequences (NC_038343, NC_014096, NC_014091, NC_014074, NC_014073, NC_038344, NC_038336, NC_014081, NC_002076, and NC_0038339) to align ORF1 gene sequences with MAFFT (37). We then used trimAl to trim our aligned sequences (38). After which, we built a neighbor-joining consensus tree, with a 1,000 bootstrap for confidence.

### Anellovirus qPCR assay

Anellovirus qPCR was performed in 25 µL reactions containing 12.5 µL of TaqMan Fast Universal PCR Master Mix (2XApplied Biosystems), 2.3 µl (10 µM) of each primer (ATV-FOR, 5′-GTGCCGIAGGTGAGTTTA-3′, and ATV- REV, 5′-AGCCCGGCCAGTCC-3′), 0.7 µL (10 µm) probe (5′ 56-FAM/TCAAGGGGCAATTCGGGCT/36-TAMSp/-3′), 4.2 µL water, and 3 µL of extracted TNA. The assay was performed with preliminary denaturation for 20 s at 95°C (slope, 4.14°C/s), followed by 40 cycles of denaturation at 95°C for 1 s (slope, 4.14°C/s), and annealing at 60°C for 20 s (slope, 3.17°C/s). A standard curve was generated using a double-stranded gene fragment cloned an anellovirus genome corresponding to genome sequence of position 1694–1756 in reference to Anellovirus complete genome GenBank accession MH649099.1. Analysis was performed with a 0.03 threshold as determined by the standard curve, measuring FAM reporter, and NFQ-MGB Quencher.

### Differential analysis

To find differentiating bacteria and viruses, we created models using R package Microbiome Multivariable Association with Linear Models (MaAsLin2) with metadata (BKV status, patient sex, and time) and controlling for longitudinal samples per patient (39). We first used all samples, to find differentiating viruses by metadata. We then stratified the samples by BK dominance and created models and reran maaslin2 in each group. We used the default *q*-value threshold of 0.25 for significance.

## Data Availability

Sequence data have been deposited to the NCBI Sequence Read Archive under accession number PRJNA587166. Code script to reproduce the analysis and plots is available in supplementary script provided on github (https://github.com/ASU-Lim-Lab/BK-Kidney/blob/e4251344ad7b44c5b5f9ce72c07c0ba1e7948809/BK_analysis_101223.R)

## SUPPLEMENTAL MATERIAL

Supplementary material spectrum.04055-23-s0001.pdf Fig. S1.

ASM does not own the copyrights to Supplemental Material that may be linked to, or accessed through, an article. The authors have granted ASM a non-exclusive, world-wide license to publish the Supplemental Material files. Please contact the corresponding author directly for reuse.
