# Supplementary material for: Longitudinal alterations in the urinary virome of kidney transplant recipients are influenced by BK viremia and patient sex
Source: Microbiol Spectr. 2024 Jun 25;12(8):e04055-23. doi: 10.1128/spectrum.04055-23 (PMC11302341; doi:10.1128/spectrum.04055-23)
Supplement: Supplementary material — Fig. S1. [file spectrum.04055-23-s0001.pdf]

A

| | Group1 | Group2 | Group3 | Group4 | Group5 | Group6 |
|---|---|---|---|---|---|---|
| Anelloviridae_12 | 0.04 | | | | | |
| Anelloviridae_5 | 0.15 | | | | | |
| Autographiviridae_1 | 0.0: | | | | | |
| Autographiviridae_2 | 0.0: | | | | | |
| Caudoviricetes_45 | 0.13 | | | | | |
| Caudoviricetes_2 | 0.05 | | | | | |
| Caudoviricetes_27 | 0.04 | | | | | |
| Caudoviricetes_4 | 0.04 | | | | | |
| Caudoviricetes_6 | 0.03 | | | | | |
| Peduoviridae_6 | 0.05 | | | | | |
| Circoviridae_14 | | 0.05 | | | | |
| Circoviridae_1 | | 0.04 | | | | |
| Circoviridae_2 | | 0.03 | | | | |
| Circoviridae_3 | | 0.03 | | | | |
| Circoviridae_4 | | 0.03 | | | | |
| Circoviridae_5 | | 0.03 | | | | |
| Circoviridae_6 | | 0.03 | | | | |
| Circoviridae_: | | 0.03 | | | | |
| Circoviridae_7 | | 0.03 | | | | |
| Circoviridae_15 | | 0.03 | | | | |
| Inoviridae_1 | | 0.04 | | | | |
| NA_10 | | 0.05 | | | | |
| Rountreeviridae_1 | | 0.03 | | | | |
| Anelloviridae_1 | | | 0.48 | 0.03 | | |
| Anelloviridae_2 | | | 0.42 | 0.03 | | |
| Peduoviridae_3 | | | | 0.05 | | |
| Microviridae_1 | | | | 0.04 | | |
| Caudoviricetes_22 | | | | 0.03 | | |
| Anelloviridae_15 | | | | 0.03 | | |
| Anaerodiviridae_1 | | | | 0.03 | | |
| Caudoviricetes_35 | | | | 0.03 | | |
| Anelloviridae_3 | | | | | 0.35 | |
| Anelloviridae_4 | | | | | 0.17 | |
| NA_9 | | | | | | 0.92 |

B

C

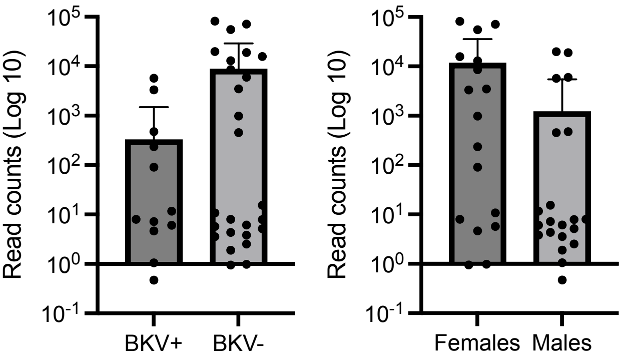


D

E

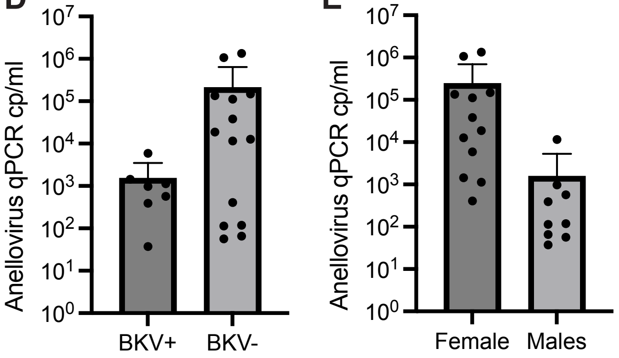


**Supplementary figure 1**. Urinary virome analysis in kidney transplant patients. (A) Average abundance of top contigs (>3%) in each community state group. (B) Anelloviridae counts by BKV status. Statistical significance assessed by mann-whitney. (C) Anelloviridae counts by patient sex. Statistical significance assessed by mann-whitney. (D) Anelloviridae qPCR copeis/ml by BKV status. Statistical significance assessed by mann-whitney. (E). Anelloviridae qPCR copeis/ml by patient sex. Statistical significance assessed by mann-whitney.
